# Unitarity or multiplicity of the psychosis: neverending question in psychopathology.

**DOI:** 10.1192/j.eurpsy.2024.1432

**Published:** 2024-08-27

**Authors:** J. J. Martínez Jambrina

**Affiliations:** Psychiatry, Hospital San Agustín, Aviles, Spain

## Abstract

**Introduction:**

- Introduce the topic of the continuity of psychoses and its relevance in contemporary psychiatry.
- Present authors as Henry EY, Jim van Os and Germán Berrios as key figures in the discussion on this topic, highlighting Ey, Dr. Jim van Os’s significant contributions to the understanding of the continuum of psychosis, Germán Berrios’s historical and cultural perspectives, and the importance of Bartolomé Llopis’s critical viewpoint. We also review the evolutionary approach about mental disorders as a keypoint in this discussion.

**Objectives:**

Analyzing and comparing the theses of Jim van Os, Ey, Germán Berrios, and Bartolomé Llopis’s critical perspective. about the continuum of the psychosis and the importance of this neverending question for its use in the clinical practice.

**Methods:**

I will present a detailed literature review and a textual analysis of their writings.

**Results:**

- Provide a brief description of Jim van Os’s theses highlighting his key ideas:
- **Dimensional Approach:** promotes a dimensional approach to understanding psychosis, viewing psychotic experiences as continuous variables rather than categorical entities. This challenges the traditional diagnostic system.
- **Psychotic-Like Experiences:** His research focuses on “psychotic-like experiences” (PLEs) in non-clinical populations, including mild hallucinations or paranoid thoughts.
- **Transdiagnostic Perspective:** His work contributes to a transdiagnostic perspective, suggesting flexibility in diagnostic boundaries in psychiatry.
- Next, introduce Germán Berrios’s theses on the same topic, emphasizing his historical and cultural perspectives and recognition of individual variability.
- Finally, introduce Bartolomé Llopis’s critical perspective:
- Llopis criticizes simplistic models of psychosis, arguing that they fail to capture the complexity of individual experiences.
- He advocates for comprehensive assessments that consider not only symptomatology but also the unique contexts and histories of individuals.
- While Ramón y Cajal is best known for his contributions to neuroscience and neuroanatomy, he did not directly apply Darwinian principles to his work in those fields. However He believed that an understanding of the evolutionary history of the brain and nervous system could provide valuable insights into their structure and function.

**Conclusions:**

- Highlight the potential for a more comprehensive and holistic approach to understanding and addressing psychotic experiences within an evolutionary context.
- The relationship between the continuum of psychosis and evolutionism is a complex and multifaceted topic. It involves exploring how the concept of the continuum of psychosis, which suggests that psychotic experiences exist on a spectrum in the general population, may relate to evolutionary theories and perspectives on mental health. We will explain some key points to consider about this and the main psychiatrists whicha dealt with this question.

**Disclosure of Interest:**

None Declared

